# Quality of life in cancer patients at the end of radiotherapy compared to a general population sample in Germany

**DOI:** 10.1002/ijc.70152

**Published:** 2025-09-12

**Authors:** Alexander Fabian, Alexander Rühle, Gregor Liegl, Justus Domschikowski, Maike Trommer, Simone Ferdinandus, Jan‐Niklas Becker, Georg Wurschi, Simon Boeke, Mathias Sonnhoff, Christoph Grott, Lukas Käsmann, Melanie Schneider, Sandra Freitag‐Wolf, Nils H. Nicolay, David Krug, Sandra Nolte

**Affiliations:** ^1^ Department of Radiation Oncology University Hospital Schleswig‐Holstein Kiel Germany; ^2^ Department of Radiation Oncology Medical Center, Faculty of Medicine, University of Freiburg Freiburg Germany; ^3^ Department of Radiation Oncology University of Leipzig Medical Center Leipzig Germany; ^4^ Comprehensive Cancer Center Central Germany, Partner Site Leipzig Leipzig Germany; ^5^ Center for Patient‐Centered Outcomes Research (CPCOR) Charité – Universitätsmedizin Berlin, Corporate Member of Freie Universität Berlin and Humboldt‐Universität zu Berlin Berlin Germany; ^6^ Department of Radiation Oncology Faculty of Medicine and University Hospital Bonn Bonn Germany; ^7^ Center for Integrated Oncology CIO Aachen Bonn Cologne Duesseldorf Cologne Germany; ^8^ Department of Radiation Oncology Olivia Newton‐John Cancer Wellness & Research Centre, Austin Health Melbourne Australia; ^9^ Department of Radiation Oncology, Cyberknife and Radiotherapy Faculty of Medicine and University Hospital Cologne Cologne Germany; ^10^ Center for Integrated Oncology Aachen Bonn Cologne Duesseldorf, CIO ABCD Cologne Germany; ^11^ Department of Radiotherapy Hannover Medical School Hannover Germany; ^12^ Department of Radiotherapy and Radiation Oncology Jena University Hospital Jena Germany; ^13^ Department of Radiation Oncology University Hospital Tübingen Tübingen Germany; ^14^ Center for Radiotherapy and Radiation Oncology Bremen Germany; ^15^ Department of Radiation Oncology University Hospital Heidelberg Heidelberg Germany; ^16^ Department of Radiation Oncology University Hospital, LMU Munich Munich Germany; ^17^ Comprehensive Pneumology Center Munich (CPC‐M), Member of the German Center for Lung Research (DZL) Munich Germany; ^18^ German Cancer Consortium (DKTK), Partner Site Munich Munich Germany; ^19^ Department of Radiotherapy and Radiation Oncology Faculty of Medicine and University Hospital Carl Gustav Carus, Technische Universität Dresden Dresden Germany; ^20^ Institute of Medical Informatics and Statistics Christian‐Albrechts‐University Kiel Kiel Germany; ^21^ Department of Radiotherapy and Radiation Oncology University Hospital Hamburg‐Eppendorf Hamburg Germany; ^22^ Person‐Centred Research Eastern Health Clinical School, Monash University Melbourne Victoria Australia

**Keywords:** general population, neoplasms, quality of life, radiotherapy

## Abstract

Germany has one of the highest cancer incidence rates in Europe. Radiotherapy is essential for patients with cancer as 50% have an evidence‐based indication for radiotherapy. However, it is unknown how health‐related quality of life (HRQoL) of cancer patients undergoing radiotherapy compares to the general population in Germany. Therefore, we conducted a secondary analysis by pooling cross‐sectional individual‐level data from a multicenter cohort of cancer patients (*n* = 1052) undergoing radiotherapy across Germany and a normative sample from the German general population (*n* = 1006). We used the EORTC QLQ‐C30 to measure global HRQoL (range: 0–100). Higher scores indicate higher HRQoL. We used ANOVA for univariable and ANCOVA with predefined covariates for multivariable analyses. As per univariable analysis, cancer patients had significantly lower global HRQoL compared with the general population (mean [*M*] = 54.6 vs. *M* = 65.9; *p* < .001). This difference was smaller but persisted in the multivariable analysis (*M* = 56.5 vs. *M* = 63.5; *p* < .001). Multivariable analyses stratified by education showed that HRQoL was only lower in cancer patients with medium (*M* = 56.2 vs. *M* = 63.0; *p* < .001) or high education (*M* = 57.0 vs. *M* = 66.5; *p* < .001) compared with the general population. The minimal important difference threshold of seven points was only met in the group with high education. In conclusion, there may be a meaningful gap in HRQoL of cancer patients at the end of radiotherapy compared with the general population, mainly in patients with higher educational levels. Upon validation, this would highlight the need for supportive care and optimized radiotherapy strategies to eventually close the HRQoL gap.

## Graphical abstract

What's new?

While radiotherapy is an essential component of oncology treatment, it remains unclear how the quality of life of treated patients compares to that of the general population. This pooled secondary analysis of two cross‐sectional studies conducted in Germany reveals existing gaps in global quality of life between the general population and patients at completion of radiotherapy, especially for patients with higher educational levels. This study supports future targeted efforts to strengthen care measures, including psychosocial counseling and symptom management, and to reduce treatment side effects to eventually close gaps in quality of life at the end of radiotherapy.

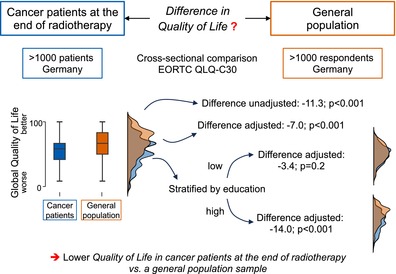

**Abbreviations**
ANCOVA: analysis of covariance
ANOVA: analysis of variance
DEGRO: German Society of Radiation Oncology
EORTC: European Organisation for Research and Treatment of Cancer
HRQoL: health‐related quality of life
MID: minimal important difference
PRO: patient‐reported outcomes
SF‐36: Short Form‐36 Health Survey

## 1 INTRODUCTION

Cancer poses a major challenge to patients as well as health care systems in Europe. Four million people were newly diagnosed with and nearly two million patients died of cancer in Europe in 2020.^1^ Among European countries, Germany has one of the highest cancer incidence rates. The EUROCARE‐6 population‐based study only recently reported that Germany ranks first for men and third for women on the complete cancer prevalence among 29 European countries.^2^ Further, the cancer incidence is projected to rise in Germany in the next several years, leading to, for example, higher rates of breast and prostate cancer.^3^

Radiotherapy is an essential component in the management of patients with cancer. In Europe, about 50% of patients with cancer have an evidence‐based indication for at least one course of radiotherapy, resulting in a large population of treated patients.^4^ A wide range of patients with cancer benefit from radiotherapy, including patients with solid tumors such as breast, prostate, head and neck, cervical, or lung cancer, or also patients with lymphoma or leukemia.^5^ However, patients treated with radiotherapy can face various challenges such as treatment side effects, perceived insecurity of treatment outcomes, or social implications of the disease itself and its treatment that may negatively impact their wellbeing. Therefore, health‐related quality of life (HRQoL) is an important patient‐centered outcome in oncology.^6^ Though various definitions exist, HRQoL is a multidimensional concept of wellbeing of patients in the context of their health that is best captured via patient‐reported outcomes (PRO), that is, outcomes directly reported by patients without interpretation by clinicians or anyone else.^7^, ^8^, ^9^ The specific concept of global HRQoL focuses on overall HRQoL and offers an overall impression of a patient's wellbeing. It is often included in Core Outcome Sets in oncology research.^10^ It can be measured using, for example, the European Organisation for Research and Treatment of Cancer (EORTC) QLQ‐C30 cancer core questionnaire, which covers 14 functioning and symptom domains in addition to said global HRQoL scale.^11^

Research into HRQoL in radiation oncology has mainly focused on specific contexts, such as distinct disease types, comparison of fractionation, or specific treatment intents.^12^, ^13^, ^14^, ^15^ Such an approach can be useful to navigate narrow research questions of specific clinical contexts. However, we lack an understanding of HRQoL across patients undergoing radiotherapy for any type of cancer (i.e., all‐comers) and to our knowledge, there is no data as to how their HRQoL compares to the HRQoL of the general population. This understanding could help to quantify potential gaps in global HRQoL, to raise awareness, and to optimize supportive measures for cancer patients receiving radiotherapy.

Therefore, we conducted a secondary analysis with pooled data from two studies performed in Germany with the aim to compare global HRQoL of cancer patients after radiotherapy with that of a general population sample.

## 2 METHODS

### 2.1 Study design and participants

Data of cancer patients after radiotherapy originated from a cross‐sectional study conducted at 11 centers in Germany, of which 10 are tertiary cancer centers. Study centers were located across all areas of Germany (Figure S1). The original study was primarily designed to investigate financial toxicity among patients treated with radiotherapy and was led by the Young German Society of Radiation Oncology (DEGRO) working group, that is, a group of early career investigators. The protocol, survey, and earlier results have been described previously.^16^, ^17^, ^18^, ^19^ The study protocol was amended to predefine the secondary analysis presented here (Data S2). In brief, patients with any type of cancer and treatment intent were invited to participate in a paper‐pencil based anonymous survey at the end of a course of radiotherapy (±2 days). Patients had to consent and had to be at least 18 years old. The recruitment period was a uniform period of 60 consecutive days from June 2022 in which all participating centers invited all potentially eligible patients. The predefined minimal participation rate was 30%. The final actual participation rate was 46% (*n* = 1075 out of *n* = 2341 eligible). Only patients with available data on global HRQoL were included in the present analysis (*n* = 1052).

Data of the German general population sample (*n* = 1006) originated from the European General Population Norm Data survey, an EORTC‐funded study on normative data of the EORTC QLQ‐C30 questionnaire published in 2019.^20^, ^21^, ^22^ In brief, data were collected via online panels across 15 countries, including Germany, with data collection outsourced to the panel research company GfK SE (www.gfk.com; GfK has since been acquired by NielsenIQ). The online panels were representative of the respective general population of a given country (i.e., limited to those with internet access), with representativeness relating to age, sex, region, hometown size, household size, and socioeconomic status. The data collection was carried out in March and April 2017. Of note, the anticipated sample size per country was *n* = 1000 participants, with sampling focusing on achieving an equal sample size in each of 10 pre‐defined age‐sex strata with a sample of *n* = 100 per stratum. As opposed to the analyses in the original publication of the general population sample, in the present analysis, we did not weigh by the respective population distribution as we compared the cohort of cancer patients with the general population sample using multivariable analysis as outlined below.^20^, ^21^, ^23^

**FIGURE 1:**
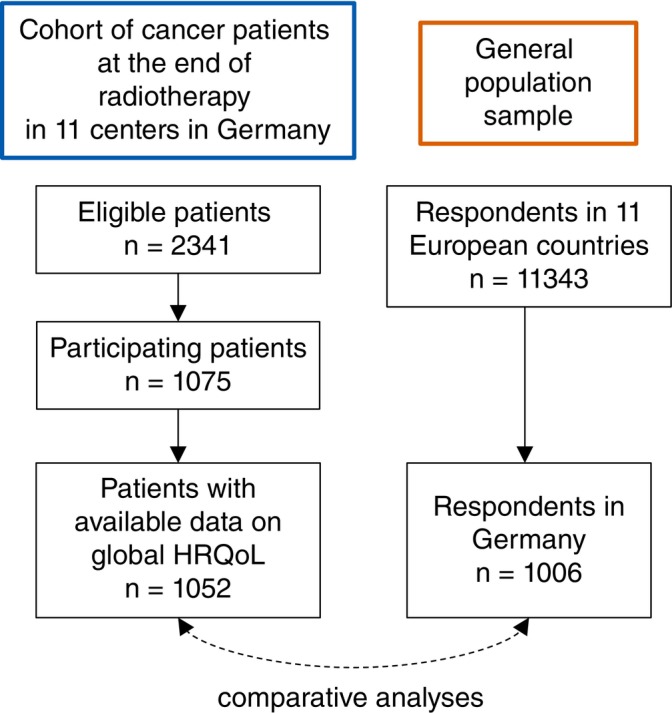
Flow chart of both study cohorts. HRQoL, health‐related quality of life.

The flow chart of both cohorts is shown in Figure 1. The STROBE guideline for reporting the analysis was applied.^24^

### 2.2 Outcomes and variables

All acquired data of cancer patients were self‐reported by patients, including patient and disease characteristics. The primary outcome for the present analysis was global HRQoL as measured by item 29 (“How would you rate your overall health during the last week?”) and item 30 (“How would you rate your overall quality of life during the last week”) of the EORTC QLQ‐C30. Both items are rated on a 7‐point scale ranging from 1 = very poor to 7 = excellent. Scores are linearly transformed to range from 0 to 100. Higher scores indicate higher global HRQoL.

Covariables that were available for the cohort of cancer patients as well as the German general population sample included age, sex, relationship status, education, and employment status. The covariables relationship status, education, and employment status were regrouped to match response categories of both cohorts as shown in the amended protocol (Data S1). We categorized education levels in low education (9 years of schooling), medium education (10 years of schooling), and high education (more than 10 years of schooling) corresponding to the German school graduations “*Hauptschulabschluss*,” “*Realschulabschluss*,” and “*Abitur*,” respectively. Another covariable was patient‐reported financial burden as per item 28 (“Has your physical condition or medical treatment caused you financial difficulties?”) of the EORTC QLQ‐C30, with scores ranging from 0 to 100 and higher scores indicating higher financial burden. Of note, no further EORTC QLQ‐C30 items than questions 28–30 were available for the cohort of cancer patients.

### 2.3 Statistical analysis

Descriptive statistics were used to describe both cohorts. Univariable one‐way Analysis of Variance (ANOVA) and Chi‐square tests were employed to detect differences between cohorts. Multivariable one‐way Analysis of Covariance (ANCOVA) analysis was used to compare mean global HRQoL of cancer patients to the general population sample, adjusting for respondent characteristics. Predefined covariates included age, sex, relationship status, employment status, and subjective financial burden. Subjective financial burden was included as a covariate because of significant associations with global HRQoL in the primary analysis of cancer patients.^16^ Cases with missing values of covariates were excluded from ANCOVA models. Effect sizes of ANCOVA analyses were reported using partial eta‐squared values (*ηp* ^2^). *ηp* ^2^ values of 0.01 to <0.06, 0.06 to 0.14, and >0.14 correspond to small, medium, and large effect size.^25^ ANCOVA model assumptions were checked visually using *Q*–*Q* plots for normality of residuals and plots of standardized deviance residuals versus fitted values for homogeneity of variance. We checked homogeneity of regression by including interaction terms of each covariate in the ANCOVA models. Tukey's post hoc test was used as appropriate. The minimal important difference (MID) threshold of global HRQoL between both cohorts was defined prior to analysis as 7 points. This was based on a recent publication by Musoro et al. that found varying MIDs across cancer types. For example, MID concerning between‐group deterioration over time of global HRQoL was reported in an anchor‐based approach to be −13 to −6 for breast cancer, −6 for prostate cancer, −4 for lung cancer, −6 for brain tumor, and −4 for head and neck cancer patients.^26^ We therefore pragmatically chose 7 points as MID, given the various cancer types included in our cohort of cancer patients. All analyses were exploratory. A two‐sided *p*‐value <.05 was considered statistically significant.

## 3 RESULTS

### 3.1 Sample characteristics

Table 1 shows the characteristics of cancer patients (*n* = 1052) at the end of a course of radiotherapy treated in Germany and the German general population sample (*n* = 1006). There were several statistically significant group differences based on ANOVA and Chi‐square tests, as appropriate: cancer patients were older (*p* < .001), less often living alone (*p* < .001), had lower educational levels (*p* < .001), were more often retired (*p* < .001), and had a higher financial burden (*p* < .001) compared with the general population sample. Breast (26%; 272/1052), prostate (19%; 195/1052), and lung cancer (10%; 102/1052) were the most common entities in the cohort of cancer patients (Table S1). Global HRQoL by the patient's self‐reported tumor entity is shown descriptively in Figure S2.

**TABLE 1:** Characteristics of cancer patients at the end of radiotherapy and of the German general population sample cohort.

	Cancer patients at end of radiotherapy	General population sample	*p*
Sample size	100% (1052)	100% (1006)	
Sex			.849
Female	49% (519)	50% (501)	
Male	51% (532)	50% (505)	
Age in years; Median (IQR)	65 (17)	55 (23)	<.001
Relationship status			<.001
Living alone	27% (286)	34% (345)	
Living with partner	72% (759)	65% (652)	
Education^a^			<.001
Less than compulsory	3% (30)	0.1% (1)	
Low	27% (288)	11% (112)	
Medium	36% (373)	39% (396)	
High	32% (340)	49% (489)	
Employment status			<.001
Employed	26% (271)	49% (494)	
Self‐employed	8% (86)	6% (60)	
Unemployed	8% (84)	10% (98)	
Retired	56% (587)	33% (333)	
Financial burden			<.001
EORTC QLQ‐C30; Mean (SD)	20.8 (28.3)	10.4 (24.1)	

*Note*: Absolute numbers are given in brackets. Numbers may not add up to 100% due to rounding error or missing values. The *p* values are based on Chi‐square test or ANOVA test depending on the variable scale.

Abbreviations: HRQoL, health‐related quality of life; IQR, interquartile range; SD, standard deviation.

^a^ Low education, medium education, and high education correspond to 9 years of schooling, 10 years of schooling, and >10 years of schooling.

### 3.2 Overall mean differences between groups

**FIGURE 2:**
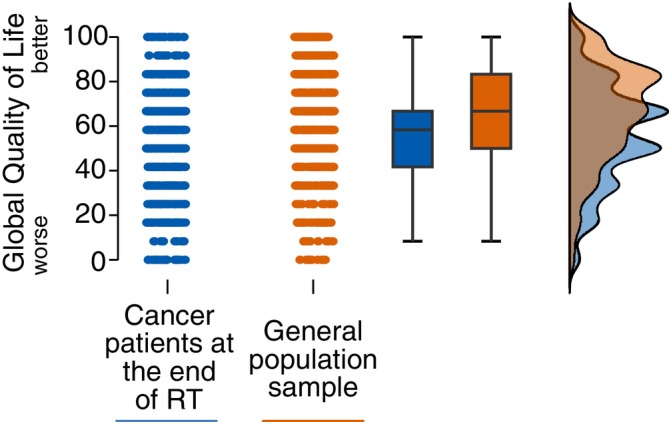
Raincloud plots of global health‐related quality of life based on the EORTC QLQ‐C30 questionnaire in cancer patients at the end of a course of radiotherapy in Germany compared with the German general population sample (“whole cohort”). Dots represent data points. Global quality of life ranges on a scale from 0 to 100, with higher values indicating higher quality of life. RT, radiotherapy.

Analyzing both whole cohorts, the unadjusted mean global HRQoL was lower in cancer patients (*n* = 1052) as compared with the general population sample (*n* = 1006) according to an ANOVA analysis: 54.6 (95% CI = 53.3–56.0) versus 65.9 (95% CI = 64.5–67.3) (mean difference −11.3; 95% CI = −13.2 to −9.4; *p* < .001), respectively. The predefined multivariable adjusted ANCOVA analysis again showed a significant difference of HRQoL in cancer patients compared with the general population sample (*F*[11936] = 42.8, *p* < .001, *ηp* ^2^ = 0.022) as visualized in Figure 2 and shown in Table S2. Marginal mean global HRQoL was 56.5 (95% CI = 55.1–58.0) in cancer patients and 63.5 (95% CI = 62.1–64.9) in the general population sample. The post hoc test suggested that global HRQoL was significantly lower in cancer patients as compared with the general population sample (mean difference −7.0; 95% CI = −9.1 to −5.9; *p* < .001). Plots on model assumption are shown in Figures S3 and S4. The model showed several statistically significant interaction terms concerning the covariate education together with age (*p* = .012), sex (*p* = .03), employment status (*p* = .028), and subjective financial burden (*p* < .001), respectively. These multiple significant interaction terms of education suggest marked inhomogeneity of regression in the predefined model, potentially limiting its validity. Therefore, we re‐ran the ANCOVA analysis on global HRQoL of cancer patients versus the general population sample stratifying by educational level including all other previously included covariates in each model. Strata were divided into respondents with low education (9 years of schooling), medium education (10 years of schooling), and high education (>10 years of schooling) according to the covariable education (Table 1). Respondents with less than compulsory education were not included in the stratified analysis due to overall low and unevenly distributed case numbers in cancer patients (3%; 30/1052) and the general population sample (0.1%; 1/1006).

### 3.3 Mean differences by educational levels

**FIGURE 3:**
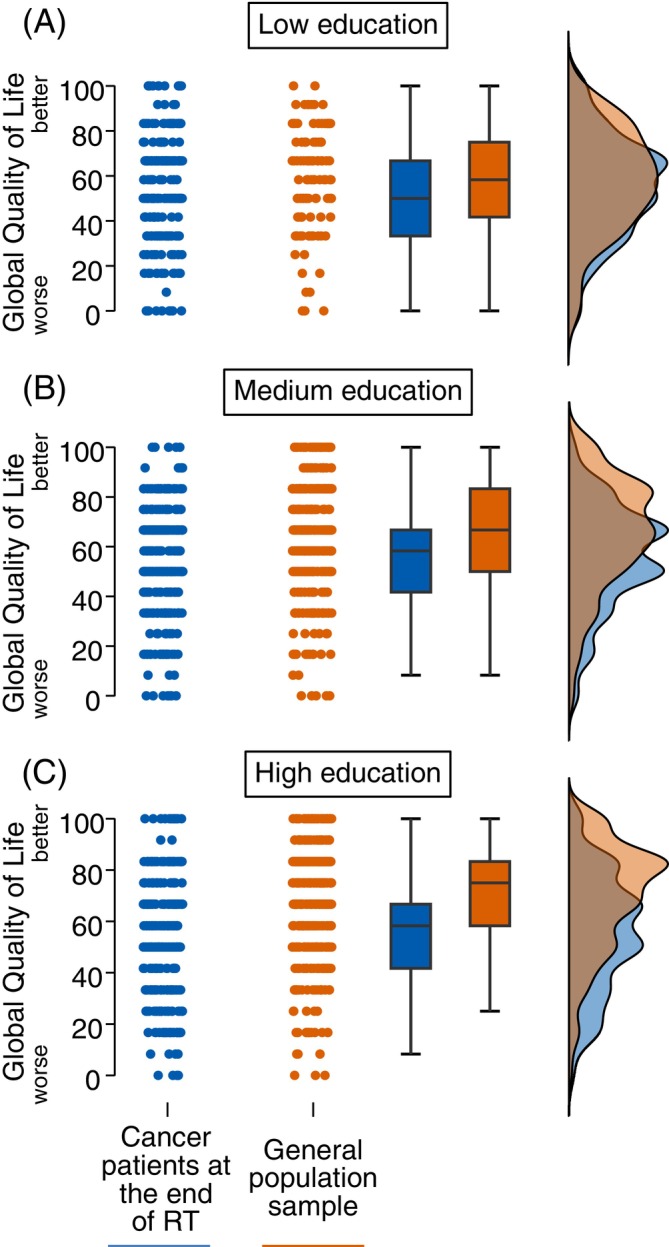
Raincloud plots of global health‐related quality of life based on the EORTC QLQ‐C30 questionnaire in cancer patients at the end of a course of radiotherapy in Germany compared with the German general population sample stratified by education level. Panel A shows respondents with low education (9 years of schooling), Panel B shows respondents with medium education (10 years of schooling), and Panel C shows respondents with high education (>10 years of schooling). Dots represent data points. Global health‐related quality of life ranges on a scale from 0 to 100, with higher values indicating higher quality of life. RT, radiotherapy.

Analyzing respondents with low education, the unadjusted mean global HRQoL was comparable between cancer patients (*n* = 288) and the general population sample (*n* = 112) as per ANOVA analysis: 54.3 (95% CI = 51.7–57.0) versus 57.7 (95% CI = 53.4–62.0) (mean difference − 3.4; *p* = .2), respectively. There was also no statistically significant difference in global HRQoL of cancer patients versus the general population sample per multivariable adjusted ANCOVA (*F*[1367] = 0.1; *p* = .7) as depicted in Figure 3A and shown in Table S3. Marginal mean global HRQoL was 55.0 (95% CI = 52.1–58.0) in cancer patients and 56.1 (95% CI = 52.6–60.5) in the general population sample (mean difference −1.1; 95% CI = −6.4 to 4.3; *p* = .7). All model assumptions were fulfilled, and there were no significant interaction terms among covariates (Figures S5 and S6).

Investigating respondents with medium education, the unadjusted mean global HRQoL was significantly lower in cancer patients (*n* = 373) compared with the general population sample (*n* = 396) as shown by ANOVA analysis: 54.9 (95% CI = 52.7–57.0) versus 64.8 (95% CI = 62.6–67.0; mean difference −9.9; *p* < .001), respectively. Global HRQoL also differed significantly in cancer patients compared with the general population sample according to the multivariable adjusted ANCOVA (*F*[1726] = 17.2; *p* < .001; *ηp* ^2^ = 0.023) as visualized in Figure 3B and shown in Table S4. Marginal mean global HRQoL was 56.2 (95% CI = 53.8–58.6) in cancer patients and 63.0 (95% CI = 60.8–65.3) in the general population sample. Post‐hoc testing showed that global HRQoL was significantly lower in cancer patients versus the general population sample (mean difference −6.9; 95% CI = −10.1 to −3.6; *p* < .001). The difference in mean global HRQoL did not meet the MID threshold of 7 points. All model assumptions were fulfilled and there were no significant interaction terms among covariates (Figures S7 and S8).

In respondents with high education, the unadjusted mean global HRQoL was again significantly lower in cancer patients (*n* = 340) compared with the general population sample (*n* = 489) per ANOVA analysis: 54.7 (95% CI = 52.3–57.2) versus 68.7 (95% CI = 66.9–70.6) (mean difference −14.0; 95% CI = −17.1 to −11.0; *p* < .001), respectively. Global HRQoL was also significantly different in cancer patients compared with the general population sample as per multivariable adjusted ANCOVA (*F*[1799] = 36.2; *p* < .001; *ηp* ^2^ = 0.043) as displayed in Figure 3C and shown in Table S5. Marginal mean global HRQoL was 57.0 (95% CI = 54.5–59.4) in cancer patients and 66.5 (95% CI = 64.5–68.4) in the general population sample. Post hoc testing demonstrated that global HRQoL was significantly lower in cancer patients versus the general population sample (mean difference −9.5; 95% CI = −12.6 to −6.4.6; *p* < .001). This difference in mean global HRQoL met the MID threshold of 7 points. All model assumptions were fulfilled except for significant interaction terms of relationship with employment status (*p* = .024) and age with relationship status (*p* = .038) (Figures S9 and S10).

Table 2 shows a summary of unadjusted as well as adjusted mean global HRQoL values and mean differences in cancer patients as compared with the general population sample and as reported above.

**TABLE 2:** Summary of mean global health‐related quality of life as measured by the EORTC QLQ‐C30 in cancer patients at the end of radiotherapy versus the German general population sample, whole cohort and stratified by low (9 years of schooling), medium (10 years of schooling), and high education (>10 years of schooling).

	Unadjusted analysis								Adjusted analysis^a^							
	Cancer patients at end of radiotherapy			General population sample					Cancer patients at end of radiotherapy			General population sample				
	*n*	*M*	95% CI	*n*	*M*	95% CI	*M* diff.	*p*	*n*	*M*	95% CI	*n*	*M*	95% CI	*M* diff.	*p*
Whole cohort	1052	54.6	53.3–56.0	1006	65.9	64.5–67.3	−11.3	<.001	974	56.5	55.1–58.0	970	63.5	62.1–64.9	−7.0	<.001
Low education	288	54.3	51.7–57.0	112	57.7	53.4–62.0	−3.4	.2	265	55.0	52.1–58.0	109	56.1	52.6–60.5	−1.1	.7
Medium education	373	54.9	52.7–57.0	396	64.8	62.6–67.0	−9.9	<.001	348	56.2	53.8–58.6	385	63.0	60.8–65.3	−6.9	<.001
High education	340	54.7	52.3–57.2	489	68.7	66.9–70.6	−14.0	<.001	331	57.0	54.5–59.4	475	66.5	64.5–68.4	−9.5	<.001

Abbreviations: CI, confidence interval; *M*, mean; *M* diff, mean difference.

^a^ ANCOVA analysis adjusting for age, sex, relationship status, education level (only for “whole cohort”), employment status, and financial burden. Marginal means are shown for the adjusted analysis. Case numbers differ from unadjusted to adjusted analyses because of the exclusion of cases with missing values of respective covariates in the adjusted analysis.

## 4 DISCUSSION

In this pooled secondary analysis of two cross‐sectional studies, we compared HRQoL in an all‐comers cohort of cancer patients at completion of radiotherapy across Germany to a general population sample. The predefined multivariable model showed a significantly lower HRQoL among cancer patients as compared with participants from the general population. Stratification in low, medium, and high education revealed that HRQoL was only significantly worse in cancer patients with medium or high education. Further, this difference in HRQoL was only clinically relevant in cancer patients with high education as per MID threshold. Effect sizes of HRQoL differences from cancer patients to the general population sample were small overall. Whilst, to our knowledge, no study has yet compared HRQoL in an all‐comers cohort of cancer patients undergoing radiotherapy to the general population, some parallels may be drawn from previous publications.

Differences in HRQoL of cancer patients in various settings as compared with the general population have been reported in previous studies. For example, Hinz et al. showed that symptom‐based HRQoL was lower in cancer patients in comparison to the general population, but global HRQoL was not.^27^ This may relate to the timing of the survey: Hinz and colleagues collected data 6 months after discharge from in‐patient rehabilitation, whereas we collected data immediately at the end of radiotherapy when acute toxicity typically peaks. This timing of our survey may have amplified the observed differences in HRQoL (i.e., worse HRQoL in cancer patients). Other studies that assessed global HRQoL at the end of radiotherapy did indeed report a reduction in HRQoL. For example, Yucel et al. conducted a monocenter longitudinal study from 2010 to 2012 and included 367 patients treated with 3D‐conformal radiotherapy.^28^ They reported a significant drop in HRQoL (mean = 63, SD = 23) as measured by the EORTC QLQ‐C30 at the end of radiotherapy as compared with follow‐up at ≥1 month (mean = 69, SD = 23) after radiotherapy. Global HRQoL at the end of radiotherapy appears higher in their cohort than in our cancer cohort, potentially due to the fact that Yucel et al. included curative cases only, whereas our cohort was not restricted to a treatment intent.^29^ Indeed, our precursor study on 100 patients at the end of radiotherapy in two centers showed a global HRQoL of 52 (SD: 23), comparable to the present analysis.^30^ Treatment‐related toxicity, emotional functioning, and uncertainty of the treatment success may be reasons for why global HRQoL appears lower at the end of treatment as compared with subsequent follow‐up.^31^, ^32^

Associations of education and health have been studied by multidisciplinary research. In fact, education is an integral part of the seminal rainbow model of Dahlgren and Whitehead on main determinants of health first published over three decades ago.^33^, ^34^ Higher levels of education correspond to higher levels of health measures, although causal mechanisms often remain unclear.^35^ More specifically, there is also data on associations of educational levels and HRQoL as measured by PRO. Concerning the general population, Teni et al. reported higher global HRQoL in respondents with higher education using the EQ‐5D‐5L in a Swedish general population sample.^36^ Using the same questionnaire in a Polish general population sample, Jankowska et al. similarly showed higher global HRQoL in respondents with higher education.^37^ Hence, there appears to be a positive association of HRQoL and education in the general population in Europe.

Concerning patients with cancer, the link of HRQoL and education might be less clear. On the one hand, a recent French observational study including over 5000 breast cancer patients reported higher HRQoL in patients with higher education based on the EORTC QLQ‐C30.^38^ This association became stronger after 1 and 2 years of follow‐up as compared with the time of initial diagnosis. Further, Annunziata et al. conducted a cross‐sectional study involving Italian survivors of various cancer types at least 5 years after diagnosis.^39^ They also found higher global HRQoL in survivors with higher education using the Short Form‐36 Health Survey (SF‐36). On the other hand, a nationwide cohort study conducted in Sweden did not find a difference in HRQoL based on education in esophageal cancer patients during follow‐up after surgery using the EORTC QLQ‐C30.^40^ Importantly, Roick et al. performed a longitudinal observational study in Germany on cancer patients with various tumor entities.^41^ Patients were included in the study at hospital admission for cancer treatment and followed up thereafter. This study did not find an association of global HRQoL and education during a follow‐up period of 6 months.

How do these findings from the literature on a clear link of HRQoL and education in the general population but less so in cancer patients compare with our results? Looking at the direction of effects of HRQoL in our study, higher global HRQoL with higher educational levels was driven by higher adjusted HRQoL in the general population. In fact, adjusted HRQoL remained nearly unchanged in cancer patients per education stratum. Therefore, our results align well with the literature. This gap in HRQoL per educational stratum may even widen further if patients were not only surveyed at treatment completion as in our study but also during follow‐up. This is based on the observation of some studies that HRQoL tends to differ in cancer patients based on educational levels during longer follow‐up or in the survivorship phase.^38^, ^39^ Finally, the relative differences of HRQoL of cancer patients at the end of radiotherapy and the German general population sample are noteworthy: cancer patients at the end of radiotherapy with high educational levels report HRQoL at a similar level to respondents from the general population sample with low educational levels, that is, while their global HRQoL shows a substantial drop after radiotherapy compared with their general population counterparts, it does not drop below the global HRQoL of the general population with low educational levels.

Our study has limitations. First, as we focused on an all‐comers approach of cancer patients undergoing radiotherapy to leverage higher level data for the field of radiotherapy as a whole, we deliberately did not differentiate by tumor site in our main analysis. Diagnoses were self‐reported and we had no information on tumor stage. Therefore, we concluded that incorporating the tumor entity into the comparison of HRQoL with the general population sample would be inappropriate and imprecise. Second, we had to predefine a regrouping of response categories of covariables to match both cohorts for analysis leading to less granularity in the data. Importantly, the general population sample coded educational levels with more detail including university degrees, whereas the cancer cohort coded educational levels only by “less than compulsory,” “9 years of school,” “10 years of school,” and “>10 years of school.” Third, additional variables unavailable in our study may have influenced patients' HRQoL, including but not limited to variables such as treatment intent, prior systemic or local therapies, or applied supportive measures. Fourth, the German general population sample was not representative of the German general population per se due to the applied sampling method by age/sex strata, as outlined in Section 2 and according publications.^20^, ^21^ Adjusting for covariables, however, allowed to compare cancer patients and the General population sample in a more meaningful way as it indeed lowered the differences of global HRQoL compared with crude analyses. Concerning the representativeness of the cancer cohort sample, the predefined participation rate was met, but still limited at 46% which may have led to non‐response bias, arguably toward higher HRQoL in responders capable to complete the survey. Fifth, we analyzed differences of global HRQoL based on potentially confounding factors such as education. Global HRQoL is measured by two out of 30 EORTC QLQ‐C30 items only. Further HRQoL domains such as specific functional domains or symptoms were not available for the cancer cohort, except for subjective financial burden, but may enrich future analyses and could potentially show differential impacts of confounding factors. Finally, the MID threshold was chosen pragmatically prior to analysis based on the distribution of cancer types and recent literature as outlined in the methods section. Higher or lower MID thresholds may have led to different conclusions. The MID threshold and its implications should therefore be interpreted cautiously.

The implication of our finding is that additional efforts are needed to further elucidate, narrow, and eventually close the gap of HRQoL in cancer patients at the end of radiotherapy in Germany compared with the general population. First, additional prospective longitudinal follow‐up studies should enrich and confirm our findings, also in the context of previous and subsequent treatments other than radiotherapy. Second, increased and tailored supportive measures should be fostered during therapy. For example, recent prospective studies have shown a positive impact of electronic PRO monitoring on PRO and HRQoL during treatment.^42^, ^43^ Third, continuous refinements of radiotherapy techniques and regimens should aim to positively impact HRQoL of patients. This could potentially be achieved, for example, by advanced radiotherapy techniques including online adaptive radiotherapy or by tailoring radiotherapy to genomic features.^44^, ^45^ Any efforts to close the gap of HRQoL in cancer patients at the end of radiotherapy, however, will need to take into account previous or concurrent treatments besides radiotherapy as well the morbidity of the cancer itself.

In conclusion, our study highlights a meaningful gap of global HRQoL in cancer patients at the end of radiotherapy treated across Germany as compared with a general population sample. However, this gap was only present in patients with higher educational levels who showed a significant drop in global HRQoL after radiotherapy compared with the general population. Upon validation of our results, future targeted efforts should aim to strengthen supportive measures, including psychosocial support and symptom management, as well as to reduce treatment side effects to eventually close gaps of global HRQoL at the end of radiotherapy.

## AUTHOR CONTRIBUTIONS

**Alexander Fabian:** Conceptualization; data curation; methodology; investigation; formal analysis; visualization; writing – original draft; project administration; software; resources. **Alexander Rühle:** Writing – review and editing; investigation. **Gregor Liegl:** Writing – original draft; conceptualization; methodology. **Justus Domschikowski:** Investigation; writing – review and editing. **Maike Trommer:** Investigation; writing – review and editing. **Simone Ferdinandus:** Investigation; writing – review and editing. **Jan‐Niklas Becker:** Investigation; writing – review and editing. **Georg Wurschi:** Writing – review and editing; investigation. **Simon Boeke:** Investigation; writing – review and editing. **Mathias Sonnhoff:** Investigation; writing – review and editing. **Christoph Grott:** Investigation; writing – review and editing. **Lukas Käsmann:** Investigation; writing – review and editing. **Melanie Schneider:** Investigation; writing – review and editing. **Sandra Freitag‐Wolf:** Methodology; writing – original draft. **Nils H. Nicolay:** Writing – review and editing. **David Krug:** Writing – review and editing. **Sandra Nolte:** Writing – review and editing; supervision; funding acquisition.

## FUNDING INFORMATION

The collection of the general population normative data was funded by the European Organisation for Research and Treatment of Cancer (EORTC) Quality of Life Group (QLG), awarded to SN, Grant Number 001 2015.

## CONFLICT OF INTEREST STATEMENT

Alexander Fabian received honoraria from Merck Sharp & Dohme outside the field of this work. Alexander Rühle received speaker fees, travel support, and research grants from Novocure, consulting fees from Johnson & Johnson, speaker fees from Merck Healthcare Germany as well as from AstraZeneca, all outside the submitted work. David Krug has received honoraria from AstraZeneca, best practice onkologie, ESO, ESMO, Gilead, med update, Merck Sharp & Dohme, Novartis, onkowissen, and Pfizer, served on advisory boards for Gilead, and has received institutional research funding from Stiftung Deutsche Krebshilfe and Merck KGaA. Justus Domschikowski received honoraria from Merck Healthcare Germany outside the field of this work. Lukas Käsmann receives an unrestricted research grant (NCT05027165) from AstraZeneca, a grant from AMGEN, and Art Tempi (MasterClass LucaNext 2023/2024); payment from German Cancer Society (for lecturing), AstraZeneca (for lecturing), and Art Tempi; and gets support for attending meetings from AstraZeneca and ELCC. Nils H. Nicolay receives consulting and speaker fees and research grants from Novocure, speaker fees and travel support from Merck Healthcare, and speaker fees from Sun Pharmaceuticals outside the submitted work. Sandra Nolte reports consulting fees and Advisory Board honoraria from the Movember Foundation and consulting fees from the Mapi Research Trust and Bertelsmann Stiftung, all outside the submitted work.

## ETHICS STATEMENT

Respective Ethics Commissions were consulted for each participating center prior to patient inclusion and approved the conduct of the study involving cancer patients (D 454/22). Additionally, an amended version of the protocol concerning the study of cancer patients was created to outline and predefine the secondary analyses presented here. This amended version was approved by the respective Ethics Commission of the medical faculty of Kiel University. The study involving cancer patients was registered prior to patient enrollment (German Clinical Trial Registry No. DRKS00028784, ARO 2022‐07).

Informed consent of cancer patients was given after potentially eligible patients read the study information sheet and subsequently returned the questionnaire on a voluntary basis. Because of the anonymous data capture, written informed consent was not undertaken per predefined study design. This approach was accepted by the respective Ethics Commissions as stated above.

Ethical approval for the EORTC European Norm Data project was not sought as the study was solely based on panel research data involving volunteers from the general population. The multinational survey conformed to the required ethical standards by obtaining informed consent from all participants and collecting data completely anonymously. Further detail is provided in the respective initial publication.^20^

## Supporting information


**Data S1.** Supporting Information.

**Data S2.** Supporting Information.

## Data Availability

Data concerning the cohort of cancer patients are available from the corresponding author upon reasonable request. Data concerning the general population sample are owned by a third party (European Organisation for Research and Treatment of Cancer). They are available upon request from the EORTC Headquarters.
