# Supplementary material for: Quality of life in cancer patients at the end of radiotherapy compared to a general population sample in Germany
Source: Int J Cancer. 2025 Sep 12;158(4):1021–30. doi: 10.1002/ijc.70152 (PMC12712360; doi:10.1002/ijc.70152)
Supplement: Supplementary file 1 — Data S1. Supporting Information. [file IJC-158-1021-s002.pdf]

# Study synopsis

| | |
|---|---|
| **Title** | **Financial Toxicity in Cancer Patients Treated with Radiotherapy – a jDEGRO Confirmatory Cross-Sectional Study** |
| Version | v1.0 21.03.2022 |
| Study coordinators | Dr. med. Alexander Fabian [1 2]<br>alexander.fabian@uksh.de; alexander.fabian@uniklinik-freiburg.de<br>Vice coordinators:<br>Dr. med. Alexander Rühle [2], alexander.ruehle@uniklinik-freiburg.de<br>Justus Domschikowski [1], justus.domschikowski@uksh.de<br>[1] Klinik für Strahlentherapie, UKSH Campus Kiel<br>Arnold-Heller-Str. 3, 24105 Kiel<br>[2] Klinik für Strahlenheilkunde, Universitätsklinikum Freiburg<br>Robert-Koch-Straße 3, 79016 Freiburg |
| Study design | Multicenter, non-interventional, confirmatory, cross-sectional study (anonymous questionnaire) |
| Study population | Inclusion criteria:<br>• Cancer patient about to complete (+/- 2d) a course of radiotherapy<br>• Ability to understand and complete the questionnaire<br>• > 18 years<br>• Patient gave informed consent<br>Exclusion criterion:<br>• Patient has already participated in the study for another course of radiotherapy |
| Patient number | At least 504 patients based on sample size calculation |
| Research questions | Primary:<br>• Confirmation of the prevalence of financial toxicity<br>• Confirmation of risk factors for financial toxicity<br>Secondary:<br>• Exploration of additional variables as risk factors for financial toxicity (e.g. distress, duration of radiotherapy)<br>• Association of financial toxicity and patient satisfaction |
| Statistics | Confirmatory for primary, and exploratory for secondary research questions |
| Data protection | Anonymous data acquisition |
| Time frame | • Data acquisition for a 60-day period from May or June 2022<br>• Data analysis until October 2022 |
| Funding | none |

# Financial Toxicity in Cancer Patients Treated with Radiotherapy – a jDEGRO Confirmatory Cross-Sectional Study

A. Fabian[1 2#], A. Ruehle[2], J. Domschikowski[1], S. Boeke[3], L. Käsmann[4], M. Trommer[5], S. Wegen[5], G. Wurschi[6], D. Krug[1], N. H. Nicolay[2]

[1]Department of Radiation Oncology, University Hospital Schleswig-Holstein, 24105 Kiel, Germany

[2]Department of Radiation Oncology, University Hospital Freiburg, 79106 Freiburg, Germany

[3]Department of Radiation Oncology, University Hospital Tübingen, Germany.

[4]LMU University Hospital, Department of Radiotherapy and Radiation Oncology, Munich

[5]University Hospital Cologne, Clinic and Policlinic for Radiation Oncology, Cyberknife and Radiotherapy, Cologne

[6]University Medical Center Jena, Department of Radiation Oncology, Jena, Germany

# Correspondence address: Alexander Fabian MD, MBA, Department of Radiation Oncology, University Hospital Freiburg, Robert-Koch-Str. 3, 79106 Freiburg, Germany, // Department of Radiation Oncology, University Hospital Schleswig-Holstein, Arnold-Heller-Str. 3, 24105 Kiel, Germany // alexander.fabian@uniklinik-freiburg.de // alexander.fabian@uksh.de

## Registration

This study will be registered prior to enrollment of the first patient in the "Open Science Framework" (www.osf.io). Furthermore, the study will be registered on the German Clinical Trials Register (DRKS) and we will apply for a registration number of the ARO (*Arbeitsgemeinschaft Radiologische Onkologie*).

## Financial Support

There is no external funding source or sponsor.

## Acknowledgment

We acknowledge statistical counseling by Martin Treppner, Institute of Medical Biometry and Statistics, University Hospital Freiburg.

## Amendments

Amendments from protocol v1.0 (21.March.2022) to protocol v2.0 (15.Feb.2025) are highlighted in yellow.

# 1 Introduction

Cancer patients face increased costs due to the disease or its treatment. Costs may be direct in terms of increased expenditures or indirect in terms of loss of income. This objective financial burden may cause subjective financial distress which in turn may result in financial toxicity (1). Financial toxicity has been linked to suboptimal treatment outcomes and patient satisfaction (2,3). Although financial toxicity has been studied extensively in the US, it has also been described in health care systems with public funding such as in Germany (4).

Radiotherapy is a major component in the treatment of cancer as approximately one out of two cancer patients receives radiotherapy at least once during the disease trajectory (5). Patients treated with radiotherapy face specific circumstances due to their treatment. This includes local side effects which may require topical supportive care which is often not reimbursed or also long treatment courses of up to 7 weeks. In this light, radiotherapy may cause both, increased direct costs as well as loss of income which may lead to financial toxicity. Yet only small exploratory studies have investigated financial toxicity in cancer patients treated with radiotherapy (Fabian et al. 2022, accepted, Strahlenther Oncol.). A better understanding of the prevalence and associated risk factors of financial toxicity is desirable to mitigate its potentially deleterious effect on treatment outcomes and patient satisfaction.

# 2 Methods

This is a cross-sectional, multi-center survey-based study. The questionnaire will be handed out to every patient meeting the eligibility criteria along with a study information. Informed consent is given if a patient returns the questionnaire in a sealed envelope. The survey is anonymous to ensure data protection.

Participating jDEGRO centers are (jDEGRO=junge DEGRO [Deutsche Gesellschaft für Radioonkologie]):

- University Hospital Freiburg, Department of Radiation Oncology, Freiburg
- University Hospital Schleswig-Holstein, Department of Radiation Oncology, Kiel
- University Hospital Cologne, Clinic and Policlinic for Radiation Oncology, Cyberknife and Radiotherapy, Cologne
- University Hospital Jena, Department of Radiotherapy and Radiation Oncology, Jena
- LMU University Hospital, Department of Radiotherapy and Radiation Oncology, Munich
- Additional German centers may follow and will be updated on the registration database DRKS

## 2.1 Eligibility criteria

Inclusion criteria are i.) cancer patient about to complete (+/- 2d) a course of radiotherapy, ii.) ability to understand and complete the questionnaire, iii.) >18 years, and iv.) patient gave informed consent.

The exclusion criterion is participation in the same study for another course of radiotherapy.

## 2.2 Outcomes and variables

All data will be retrieved from a questionnaire. The questionnaire was pilot tested on five voluntary, potentially eligible patients in March 2022. Thereafter, subtle changes to the layout were implemented. Furthermore, an additional sex category ("diverse") was added.

Outcome data originate from two questions on objective financial burden (additional costs and loss of income in the context of radiotherapy), one question on subjective financial distress (question 28 of the EORTC QLQ-C30), and a single question on patient satisfaction with radiotherapy (Numeric Rating Scale 0-10, modified based on (6)). At the conception of this study, there was no validated German measure to assess financial toxicity in terms of a single effect size. Therefore, we relied on subjective financial distress as surrogate parameter for financial toxicity as in a previous study (Fabian et al. 2022, accepted, Strahlenther Oncol.).

Variables include sociodemographic data (sex, age, marital status, education level, health insurance, exemption from copayments in social health insurance), data on the disease and radiotherapy (type of cancer, duration of radiotherapy, concomitant chemotherapy, out- vs. inpatient), data on occupation and finances (employment status, net household income), patient

distress (NRS distress thermometer (7)), and overall health-related quality of life (question 29 and 30 of the EORTC QLQ-C30).

## *2.3 Statistical analysis plan*

One primary research question is the confirmation of the overall rate of financial toxicity per subjective financial burden (question 28 EORTC QLQ-C30) as detected in a previous exploratory study (Fabian et al. 2022, accepted, Strahlenther Oncol.). This study employed the same eligibility criteria. The questionnaire from this study was only slightly modified for the present study. Questions on outcomes or variables for the primary research questions were not modified. In the previous study, overall 31% of the patients reported subjective financial distress (Fabian et al. 2022, accepted, Strahlenther Oncol.).
To confirm this overall prevalence of financial subjective distress, we determined a sample size of n = 329 based on the expected prevalence of 31% and assuming a precision of the estimate of 5%. Accordingly, if the prevalence of subjective financial distress falls into the 95% binomial exact confidence interval of 26.04-36.31%, this primary research question will be regarded as confirmed. The subgrades of subjective financial distress were rated by patients as 21% “a little”, 6% “quite a bit”, and 4% “very much” in the previous study. Yet of note, we considered the endpoint to be binary (absence/presence of financial distress) to determine the necessary sample size for estimating the prevalence.

Another primary research question is the confirmation of risk factors of financial toxicity per subjective financial distress as shown in the previous study (Fabian et al. 2022, accepted, Strahlenther Oncol.). Statistically significant risk factors included lower net household income (OR= 0.28; p = 0.01), higher direct costs (OR= 2.78; p = 0.021), and higher loss of income (OR= 2.48 p = 0.025) per multivariate ordinal regression. Active employment status (OR= 16.96; p = 0.067) and lower health-related quality of life (OR= 0.51; p = 0.058) were also associated with higher subjective financial distress per multivariate ordinal regression.
To be able to confirm these risk factors, we conducted a sample size calculation based on a logistic regression model using G*Power Version 3.1.9.6 (Faul et al. 2007,2009) since sample size determination for ordinal regression models requires potentially complex simulation approaches (8,9). We used a conservatory approach by calculating the sample size for the smallest response subcategory of the dependent variable (=”very much” subjective financial distress), which was reported by 4% of the patients based on the previous study as mentioned above. We conducted the sample size calculation based on a type I error of $\alpha = 0.05$, a power of 0.8, an odds ratio for the expected effect size of 0.5, and a correction factor for covariates (for details, see G * Power 3.1 manual, p. 70, ‘$R^2$ other X’), the resulting sample size was n = 504 patients for a two-sided z-test. If the risk factors mentioned above significantly predict the extent of subjective financial distress per ordinal regression analysis, these risk factors will then be regarded as confirmed in the setting of our study.

Secondary research questions include the exploration of additional variables as risk factors for financial toxicity (e.g. distress, duration of radiotherapy) as well as the exploration of an association of subjective financial distress and patient satisfaction.

Participation rate will be described based on returned questionnaires and the number of cancer patients that finished a course of radiotherapy during the study period. This approach underestimates the participation rate as also potentially ineligible patients (e.g. unable to understand questionnaire) will be included in the latter number. However, this approach appears reasonably feasible due to the large overall patient numbers in each center.

## 2.4 Duration of the study

To achieve the largest planned sample size of 504 patients we plan patient accrual during two consecutive months. This assumption is based on five participating centers, 2.000 treated patients per year per center, and a participation rate of 30% resulting in 600 patients after 60 days of accrual. The participation rate of 30% is a conservatory estimate based on the previous study (53.3%) and based on the underestimating definition of participation rate (as mentioned in chapter 2.3).
In case the planned sample size will be achieved earlier, accrual will continue to complete two months of accrual in order to facilitate additional exploratory analyses. In case the planned sample size should not be achieved after two months, accrual will continue until the planned sample size will be achieved if feasible in a reasonable amount of time. All centers will report the number of included patients on a weekly basis to the study coordinators.

# 3 Ethical aspects and data protection

## *3.1 Ethical and legal issues*

We do not expect any ethical or legal issues in the context of this study due to the non-interventional, cross-sectional, and anonymous study design. Study participation is voluntary. Participation or non-participation will not influence a patients' care by any means. Patient insurance is not necessary for the purpose of this study as no intervention other than the questionnaire administration at a routine visit will be administered.

The Ethics Committee of the Medical Faculty of Christian-Albrecht-University of Kiel will be asked for approval of the study prior to enrolment of the first patient. Each participating center will apply for approval of the respective Ethics Committee prior to enrolment of the first patient at their site.

## *3.2 Data protection and data handling*

We do not expect any issues concerning data protection as all data retrieved within the study is anonymous due to the large number of potentially eligible patients (approx. over 2.000 patients treated in each radiotherapy department per year). There will not be any form of pseudonymization. Therefore, it will not be possible to link data to an individual patient.

As described above, the questionnaire and a patient information sheet will be handed out to eligible patients. Therefore, informed consent is given if a patient returns the questionnaire in a sealed envelope to the hospital staff. At the end of the study, the pooled envelopes will be opened only by members of the local study team. The anonymous data arising from the questionnaires will be extracted into an Excel file in each participating center. The Excel file will be protected by a password given by the local study team. Participating centers will send the Excel file via mail to the study coordinators. The passwords will be transmitted via phone call. The data of all centers will be pooled and analyzed by the study coordinators.

Due to the anonymous character of data in this study, the EU General Data Protection Regulation/ *Datenschutz-Grundverordnung* (GDPR / *DSGVO*) does not apply per Recital 26 GDPR / *Erw.Gr. 26 DSGVO*.

# 4 Publication

We seek publication of results of this study. A priori, each participating center is respected for co-authorship (1 co-author) if at least 50 patients have been recruited in the respective center. Additional co-authors may be respected per authorship guideline of the jDEGRO if feasible.

# 5 Secondary analysis of global quality of life in comparison to general population data

## 5.1 Outline

Every other patient with cancer has an evidence-based indication for radiotherapy (10). The cancer disease itself or its treatment may negatively impact a patient's health-related quality of life (HRQoL). To measure HRQoL, patient-reported outcome measures (PROMs) are commonly used, for example in the form of validated questionnaires (11). These questionnaires may either investigate symptoms, functioning, or global HRQoL or each of these. For example, the generic PROM of the European Organization for Research and Treatment of Cancer (EORTC), the cancer core QLQ-C30, is commonly used in oncology (12).

Research on quality of life has recently rightfully focused on cancer survivors long after completion of treatment (13). Hinz and colleagues, for example, surveyed cancer patients at least 6 months after treatment and did not find a difference in global quality of life as compared to the general population (14). Yet patients could be exposed to decreased global quality of life at the end of a specific treatment such as radiotherapy despite significant advances in treatment techniques. More specifically, we lack a good understanding of overall quality of life of cancer patients in representative cohorts across disease types at the end of radiotherapy. This understanding could help to raise awareness for global quality of life and might underline the need for intensified supportive measures and improvements in treatment techniques.

The study of this protocol recruited patients in a coherent cross-sectional timeframe and included 11 centers across Germany resulting in over 1,000 participating patients. These patients also rated their global quality of life based on the EORTC QLQ-C30 (question 29+30) along with general covariables. Nolte and colleagues published general population normative data on the EORTC QLQ-C30 in 2019. The dataset includes responses from over 1,000 individuals in Germany and has important general covariables.

Therefore, the primary aim of this secondary analysis is to compare global quality of life of cancer patients at the end of radiotherapy with the general population.

## 5.2 Methods

This is a secondary analysis with pooled data from two studies. Data from cancer patients at the end of a course of radiotherapy will be used as collected for the primary study of this protocol. In total, 1075 patients were recruited of which 1052 responded to global quality of life. The general population cohort, including 1006 individuals in Germany, is based on Nolte et al. (15).

### *5.2.1 Outcomes and variables*

The primary outcome of this analysis is global quality of life based on question 29 and question 30 of the EORTC QLQ-C30 in cancer patients at the end of radiotherapy as compared to data

form a general population. Global quality of life will be calculated as suggested in the scoring manual of the questionnaire.

Covariables include general patient/individual characteristics including sex, age, education level, employment status, relationship status, and financial difficulties based on question 28 of the EORTC QLQ-C30.

Following groups will be applied to account for differences in categories of covariables in the cohort of cancer patients and general population cohort:

| **Covariable** | **Cancer patients** | **General population** | **Present analysis** |
|---|---|---|---|
| **Sex** | Male | Male | Male |
| | Female | Female | Female |
| **Age** | continuous | continuous | continuous |
| **Education** | None | Less than compulsory | Less than compulsory |
| | < 10 years of education *(„Hauptschulabschluss“)* | Compulsory | < 10 years of education *(„Hauptschulabschluss“)* |
| | 10 years of education *(„Realschulabschluss“)* | Some post compulsory | 10 years of education *(„Realschulabschluss“)* |
| | > 10 years of education *(„Abitur“)* | Post compulsory below university | > 10 years of education *(„Abitur“)* |
| | | University degree | > 10 years of education *(„Abitur“)* |
| | | Postgraduate degree | > 10 years of education *(„Abitur“)* |
| | | prefer not to answer | missing |
| **Employment status** | Employed | Employed full-time | Employed |
| | | Employed part-time | Employed |
| | Unemployed | Unemployed | Unemployed |
| | | Student | Unemployed |
| | | Homemaker | Unemployed |
| | Retired | Retired | Retired |
| | Self-employed | Self-employed | Self-employed |
| | | Other | Variable transformed according to free text entries if feasible, otherwise missing |
| | | Prefer not to answer | missing |
| | Official | | Employed |
| **Relationship status** | Lives alone | Single | Lives alone |
| | | Separated/divorced/widowed | Lives alone |
| | Lives with partner | Married or in steady relationship | Lives with partner |
| | | Prefer not to answer | missing |

### *5.2.2 Statistical analysis plan*

Cohort characteristics will be displayed by descriptive statistics.

To analyze potential differences in global quality of life in both cohorts (cancer patients vs. general population) an ANOVA test will be performed for univariable and an ANCOVA test

will be used for multivariable analysis controlling for general patient/individual characteristics. Covariables in the ANCOVA test will include age, sex, relationship status, employment status, and subjective financial burden based on question 28 of the EORTC QLQ-C30 questionnaire. These covariables were chosen because of their potential influence on global quality of life and because of availability in both data sets.

A minimally important difference of 7 points of global quality of life in cancer patients compared to the general population will be considered as clinically meaningful (16). This falls into the range of 5 – 10 points which is often used as threshold and may reflect a compromise within this range as the cohort for cancer patients will consist of various different disease types.

## *5.3 Ethical aspects and data protection in the context of the secondary analysis*

We do not expect any ethical or legal issues in the context of this analysis as it is a secondary analysis without the need for any further patient accrual. The Ethics Committee of the Medical Faculty of Christian-Albrecht-University of Kiel will be asked for approval of the protocol amendment to include this secondary analysis.

Importantly, all patients in both “financial toxicity studies” participated anonymously. All study data is anonymous and no further information can be retrieved from medical records as no pseudonyms exist. Due to the anonymous nature of this data, the *Datenschutz-Grundverordnung* (GDPR / *DSGVO*) does not apply. The EORTC approved data sharing concerning the general population data of the EORTC QLQ-C30 questionnaire.

## *5.4 Authorship*

Alexander Fabian will be first author of a potential publication. Sandra Nolte will be last author. Further co-authors will be respected as applicable and limitations allow.

# 6 References

1. Witte J, Mehlis K, Surmann B, Lingnau R, Damm O, Greiner W, u. a. Methods for measuring financial toxicity after cancer diagnosis and treatment: a systematic review and its implications. Annals of Oncology. 2019;30(7):1061–70.

2. Yabroff KR, Bradley C, Shih YCT. Understanding Financial Hardship Among Cancer Survivors in the United States: Strategies for Prevention and Mitigation. Journal of Clinical Oncology [Internet]. 2019 [zitiert 1. November 2021]; Verfügbar unter: https://ascopubs.org/doi/pdf/10.1200/JCO.19.01564

3. Perrone F, Jommi C, Maio MD, Gimigliano A, Gridelli C, Pignata S, u. a. The association of financial difficulties with clinical outcomes in cancer patients: secondary analysis of 16 academic prospective clinical trials conducted in Italy. Annals of Oncology. 2016;27(12):2224–9.

4. Longo CJ, Fitch MI, Banfield L, Hanly P, Yabroff KR, Sharp L. Financial toxicity associated with a cancer diagnosis in publicly funded healthcare countries: a systematic review. Supportive Care in Cancer. Oktober 2020;28(10):4645–65.

5. Lievens Y, Borras JM, Grau C. Provision and use of radiotherapy in Europe. Molecular Oncology. 2020;14(7):1461–9.

6. Gomez-Cano M, Lyratzopoulos G, Abel GA. Patient Experience Drivers of Overall Satisfaction With Care in Cancer Patients: Evidence From Responders to the English Cancer Patient Experience Survey. J Patient Experience. 1. Oktober 2020;7(5):758–65.

7. Mehnert A, Müller D, Lehmann C, Koch U. Die deutsche Version des NCCN Distress-Thermometers: Empirische Prüfung eines Screening-Instruments zur Erfassung psychosozialer Belastung bei Krebspatienten. Zeitschrift für Psychiatrie Psychologie und Psychotherapie. 1. Januar 2006;54:213–23.

8. Faul F, Erdfelder E, Lang AG, Buchner A. G*Power 3: A flexible statistical power analysis program for the social, behavioral, and biomedical sciences. Behavior Research Methods. 1. Mai 2007;39(2):175–91.

9. Faul F, Erdfelder E, Buchner A, Lang AG. Statistical power analyses using G*Power 3.1: Tests for correlation and regression analyses. Behavior Research Methods. 1. November 2009;41(4):1149–60.

10. Lievens Y, Borras JM, Grau C. Provision and use of radiotherapy in Europe. Mol Oncol. Juli 2020;14(7):1461–9.

11. LeBlanc TW, Abernethy AP. Patient-reported outcomes in cancer care - hearing the patient voice at greater volume. Nat Rev Clin Oncol. Dezember 2017;14(12):763–72.

12. Aaronson NK, Ahmedzai S, Bergman B, Bullinger M, Cull A, Duez NJ, u. a. The European Organization for Research and Treatment of Cancer QLQ-C30: a quality-of-life instrument for use in international clinical trials in oncology. J Natl Cancer Inst. 1993;85(5):365–76.

13. Vaz-Luis I, Masiero M, Cavaletti G, Cervantes A, Chlebowski RT, Curigliano G, u. a. ESMO Expert Consensus Statements on Cancer Survivorship: promoting high-quality

survivorship care and research in Europe. Annals of Oncology. 1. November 2022;33(11):1119–33.

14. Hinz A, Mehnert A, Dégi C, Reissmann DR, Schotte D, Schulte T. The relationship between global and specific components of quality of life, assessed with the EORTC QLQ-C30 in a sample of 2019 cancer patients. Eur J Cancer Care (Engl). März 2017;26(2).

15. Nolte S, Liegl G, Petersen MA, Aaronson NK, Costantini A, Fayers PM, u. a. General population normative data for the EORTC QLQ-C30 health-related quality of life questionnaire based on 15,386 persons across 13 European countries, Canada and the Unites States. European Journal of Cancer. 1. Januar 2019;107:153–63.

16. Musoro JZ, Coens C, Sprangers MAG, Brandberg Y, Groenvold M, Flechtner HH, u. a. Minimally important differences for interpreting EORTC QLQ-C30 change scores over time: A synthesis across 21 clinical trials involving nine different cancer types. European Journal of Cancer. 1. Juli 2023;188:171–82.

## Signature

15.Feb.2025 ____________________________

Study coordinator Dr. Alexander Fabian, MBA
