# Supplementary material for: Quality of life in cancer patients at the end of radiotherapy compared to a general population sample in Germany
Source: Int J Cancer. 2025 Sep 12;158(4):1021–30. doi: 10.1002/ijc.70152 (PMC12712360; doi:10.1002/ijc.70152)

Quality of life in cancer patients at the end of radiotherapy compared a general population sample in Germany

Alexander Fabian, Alexander Rühle, Gregor Liegl, Justus Domschikowski, Maike Trommer, Simone Ferdinandus, Jan-Niklas Becker, Georg Wurschi, Simon Boeke, Mathias Sonnhoff Christoph Grott, Lukas Käsmann, Melanie Schneider, Sandra Freitag-Wolf, Nils H. Nicolay, David Krug, Sandra Nolte

Supplementary Material

Supplementary Material	Page
**Study Protocol** – provided as separate pdf file	-
**Supplementary Figure 1** Map of participating radiotherapy study centers across Germany that enrolled cancer patients.	p 2
**Supplementary Table 1** Tumor entities in the cohort of cancer patients at the end of a course of radiotherapy in Germany (n = 1052). Numbers may not add up to 100% due to rounding error or missing values	p 2
**Supplementary Figure 2** Global Health-Related Quality of Life (HRQoL) in cancer patients at the end of radiotherapy by patient-reported tumor entity based on the EORTC QLQ-C30 questionnaire.	p 3
**Supplementary Table 2** Adjusted multivariable ANCOVA model of global Health-related Quality of Life based on the EORTC QLQ-C30 in cancer patients (n = 1052) at the end of a course of radiotherapy compared to a general population sample (n = 1006) in Germany ("**whole cohort**")	p 4
**Supplementary Figure 3** Q-Q plot of residuals to assess normality of residuals corresponding to the model shown ("**whole cohort**") in supplementary Table 2	p 4
**Supplementary Figure 4** Plot of standardized deviance residuals versus fitted values to assess homogeneity of variance corresponding to the model shown ("**whole cohort**") in Supplementary Table 2	p 4
**Supplementary Table 3** Adjusted multivariable ANCOVA model of global Health-related Quality of Life based on the EORTC QLQ-C30 in respondents with **low education**. Cancer patients (n = 288) at the end of a course of radiotherapy were compared to a general population sample (n = 112) in Germany	p 5
**Supplementary Figure 5** Q-Q plot of residuals to assess normality of residuals corresponding to the model shown ("**low education**") in supplementary Table 3	p 5
**Supplementary Figure 6** Plot of standardized deviance residuals versus fitted values to assess homogeneity of variance corresponding to the model shown ("**low education**") in Supplementary Table 3	p 5
**Supplementary Table 4** Adjusted multivariable ANCOVA model of global Health-related Quality of Life based on the EORTC QLQ-C30 in respondents with **medium education**. Cancer patients (n = 373) at the end of a course of radiotherapy were compared to a general population sample (n = 396) in Germany. Abbreviation: ηp^2, partial eta-squared	p 6
**Supplementary Figure 7** Q-Q plot of residuals to assess normality of residuals corresponding to the model shown ("**medium education**") in supplementary Table 4	p 6
**Supplementary Figure 8** Plot of standardized deviance residuals versus fitted values to assess homogeneity of variance corresponding to the model shown ("**medium education**") in Supplementary Table 4.	p 6
**Supplementary Table 5** Adjusted multivariable ANCOVA model of global Health-related Quality of Life based on the EORTC QLQ-C30 in respondents with **high education**. Cancer patients (n = 340) at the end of a course of radiotherapy were compared to a general population sample (n = 489) in Germany	p 7
**Supplementary Figure 9** Q-Q plot of residuals to assess normality of residuals corresponding to the model shown ("**high education**") in supplementary Table 5	p 7
**Supplementary Figure 10** Plot of standardized deviance residuals versus fitted values to assess homogeneity of variance corresponding to the model shown ("**high education**") in Supplementary Table 5	p 7

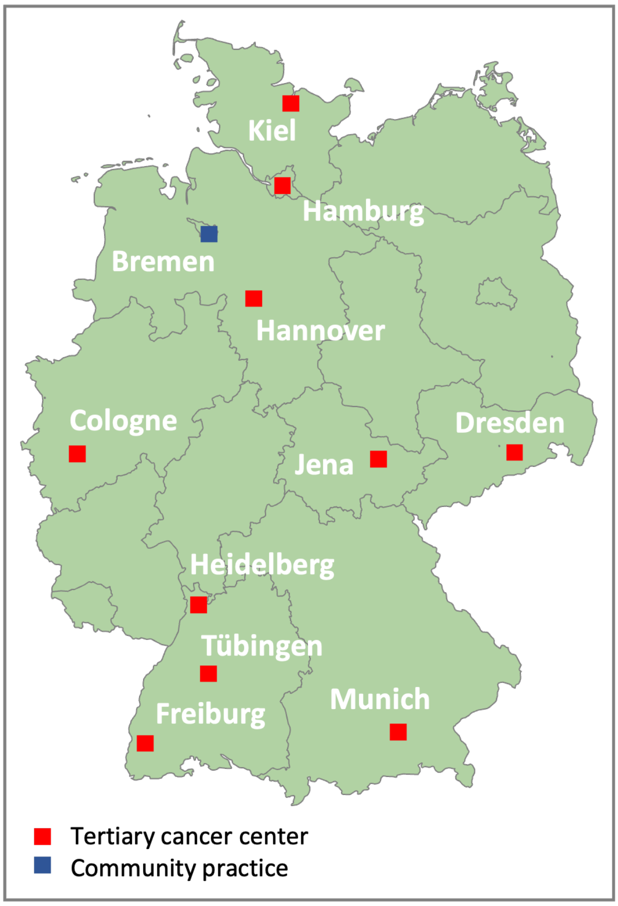

Supplementary Figure 1 Map of participating radiotherapy study centers across Germany that enrolled cancer patients.

Supplementary Table 1 Tumor entities in the cohort of cancer patients at the end of a course of radiotherapy in Germany (n = 1052). Numbers may not add up to 100% due to rounding error or missing values

**Tumor entity**	**% (n)**
Breast cancer	26% (272)
Prostate cancer	19% (195)
Lung cancer	10% (102)
Brain tumor (primary or secondary)	8% (79)
Head and neck cancer	7% (74)
Gynecological cancer	4% (37)
Rectal cancer	3% (35)
Other	22% (234)

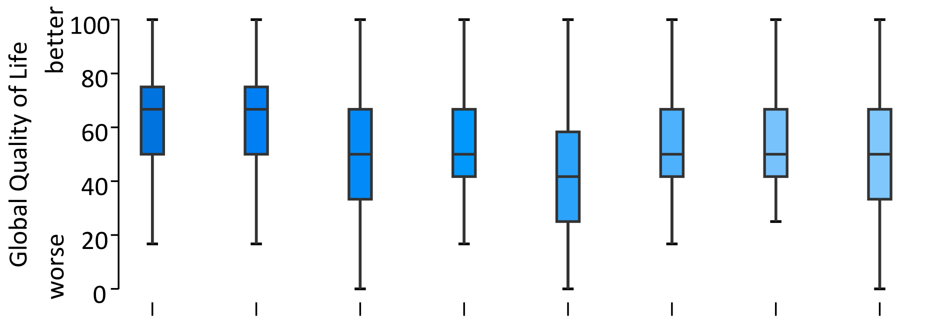

	Breast	Prostate	Lung	Brain	H&N	Gyn	Rectal	Other
Median	66.7	66.7	50.0	50.0	41.7	50.0	50.0	50.0
IQR	25.0	25.0	33.4	25.0	33.3	25.0	25.0	33.4
Mean	58.9	59.2	54.2	52.6	42.5	52.3	54.5	51.3
SD	21.3	21.2	23.1	21.0	24.3	23.4	22.5	21.2

Supplementary Figure 2 Global Health-Related Quality of Life (HRQoL) in cancer patients at the end of radiotherapy by patient-reported tumor entity based on the EORTC QLQ-C30 questionnaire. Global HrQoL ranges on a scale from 0 to 100 with higher values indicating higher HRQoL. Abbreviations: Gyn, gynecological; H&N, head and neck; IQR, interquartile range

Supplementary Table 2 Adjusted multivariable ANCOVA model of global Health-related Quality of Life based on the EORTC QLQ-C30 in cancer patients (n = 1052) at the end of a course of radiotherapy compared to a general population sample (n = 1006) in Germany ("**whole cohort**"). Abbreviation: ηp^2, partial eta-squared

Dependent variable	Global Health-related Quality of life			
		F	p	ηp^2
Fixed factor	Cancer patients vs. general population	42.8	< 0.001	0.022
Covariates	Age	3.4	0.065	0.002
	Sex	0.002	1.0	1.3×10^{-6}
	Relationship status	1.8	0.2	$9.1\text{x}10^{-4}$
	Education	1.9	0.164	$10\text{x}10^{-4}$
	Employment status	4.0	0.046	0.002
	Financial burden	201.7	< 0.001	0.094

Supplementary Figure 3 Q-Q plot of residuals to assess normality of residuals corresponding to the model shown ("**whole cohort**") in supplementary Table 2

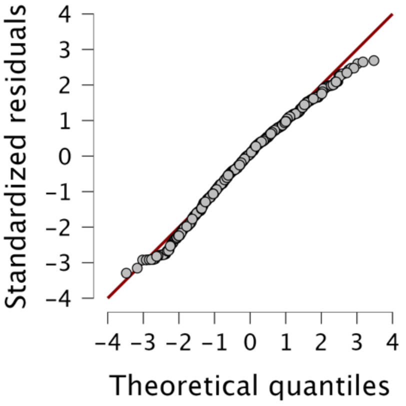

Supplementary Figure 4 Plot of standardized deviance residuals versus fitted values to assess homogeneity of variance corresponding to the model shown ("**whole cohort**") in Supplementary Table 2. Note that this graph was calculated using a generalized linear model parallel to the respective ANCOVA model

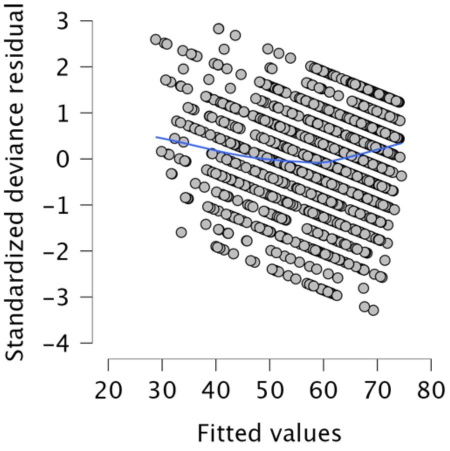

Supplementary Table 3 Adjusted multivariable ANCOVA model of global Health-related Quality of Life based on the EORTC QLQ-C30 in respondents with **low education**. Cancer patients (n = 288) at the end of a course of radiotherapy were compared to a general population sample (n = 112) in Germany. Abbreviation: ηp^2, partial eta-squared

Dependent variable	Global Health-related Quality of life			
		F	p	ηp^2
Fixed factor	Cancer patients vs. general population	0.1	0.7	3.9×10^{-4}
Covariates	Age	0.002	1.0	6.5×10^{-6}
	Sex	1.9	0.2	0.005
	Relationship status	0.002	1.0	5.1×10^{-6}
	Employment status	1.4	0.2	0.004
	Financial burden	19.5	< 0.001	0.05

Supplementary Figure 5 Q-Q plot of residuals to assess normality of residuals corresponding to the model shown (“**low education**”) in supplementary Table 3

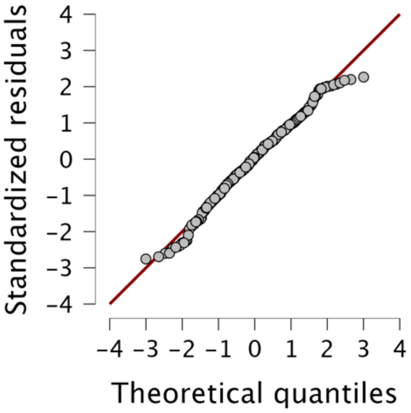

Supplementary Figure 6 Plot of standardized deviance residuals versus fitted values to assess homogeneity of variance corresponding to the model shown (“**low education**”) in Supplementary Table 3. Note that this graph was calculated using a generalized linear model parallel to the respective ANCOVA model

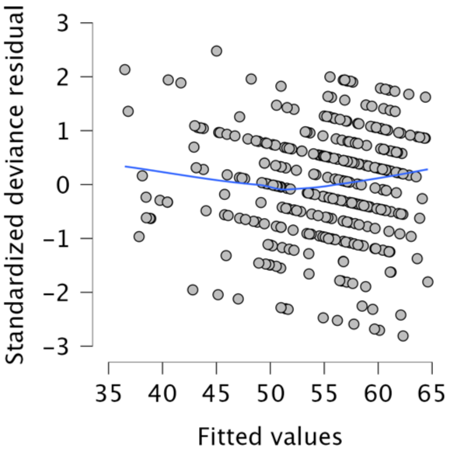

Supplementary Table 4 Adjusted multivariable ANCOVA model of global Health-related Quality of Life based on the EORTC QLQ-C30 in respondents with **medium education**. Cancer patients (n = 373) at the end of a course of radiotherapy were compared to a general population sample (n = 396) in Germany. Abbreviation: ηp^2, partial eta-squared

Dependent variable	Global Health-related Quality of life			
		F	p	ηp^2
Fixed factor	Cancer patients vs. general population	17.2	< .001	0.023
Covariates	Age	0.01	0.9	1.4×10^{-5}
	Sex	0.3	0.6	3.8×10^{-4}
	Relationship status	0.7	0.4	9.8×10^{-4}
	Employment status	4.2	0.041	0.006
	Financial burden	65.8	< .001	0.083

Supplementary Figure 7 Q-Q plot of residuals to assess normality of residuals corresponding to the model shown (“**medium education**”) in supplementary Table 4

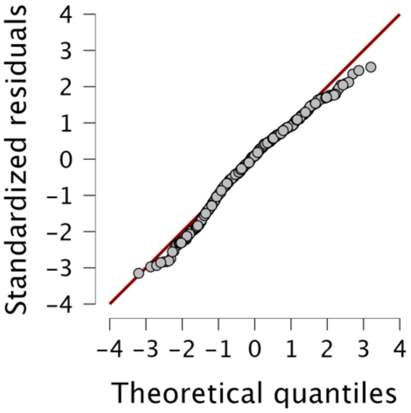

Supplementary Figure 8 Plot of standardized deviance residuals versus fitted values to assess homogeneity of variance corresponding to the model shown (“**medium education**”) in Supplementary Table 4. Note that this graph was calculated using a generalized linear model parallel to the respective ANCOVA model

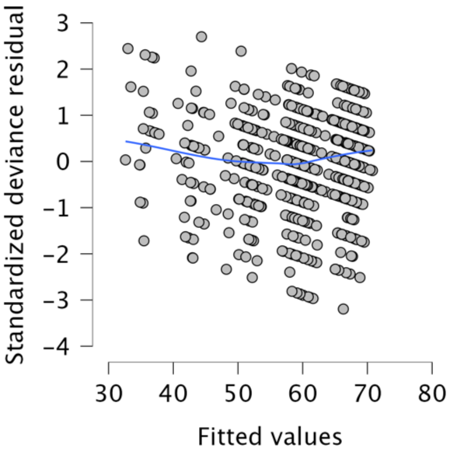

Supplementary Table 5 Adjusted multivariable ANCOVA model of global Health-related Quality of Life based on the EORTC QLQ-C30 in respondents with **high education**. Cancer patients (n = 340) at the end of a course of radiotherapy were compared to a general population sample (n = 489) in Germany. Abbreviation: ηp^2, partial eta-squared

Dependent variable	Global Health-related Quality of life			
		F	p	ηp^2
Fixed factor	Cancer patients vs. general population	36.2	< .001	0.043
Covariates	Age	7.5	0.006	0.009
	Sex	1.4	0.3	0.002
	Relationship status	1.8	0.2	0.002
	Employment status	0.1	0.7	1.7×10^{-4}
	Financial burden	138.3	< .001	0.148

Supplementary Figure 9 Q-Q plot of residuals to assess normality of residuals corresponding to the model shown ("**high education**") in supplementary Table 5

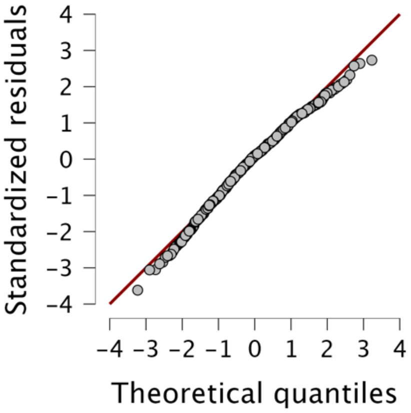

Supplementary Figure 10 Plot of standardized deviance residuals versus fitted values to assess homogeneity of variance corresponding to the model shown ("**high education**") in Supplementary Table 5. Note that this graph was calculated using a generalized linear model parallel to the respective ANCOVA model

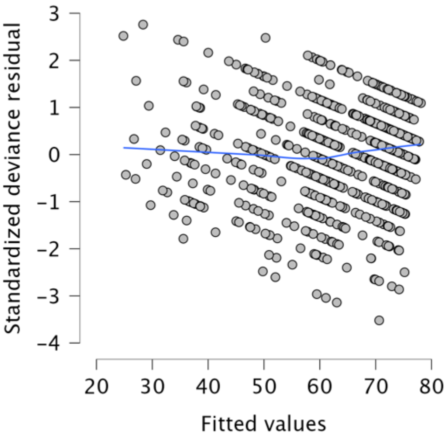

Supplement: Supplementary file 2 — Data S2. Supporting Information. [file IJC-158-1021-s001.pdf]
